# Current Status of and Future Prospects for Drug-Eluting Stents and Scaffolds in Infrapopliteal Arteries

**DOI:** 10.3390/jcm13061757

**Published:** 2024-03-19

**Authors:** Elizabeth Lim, Ramon L. Varcoe

**Affiliations:** 1 The Prince of Wales Hospital, Sydney, NSW 2031, Australia; elizabeth.lim4@health.nsw.gov.au; 2 The Prince of Wales Hospital, University of New South Wales, Sydney, NSW 2031, Australia

**Keywords:** critical limb-threatening ischaemia, drug-eluting stents, endovascular, bioresorbable scaffold

## Abstract

**Background:** Chronic limb-threatening ischaemia can be a debilitating disease and may result in limb amputation if untreated. Atherosclerotic disease of the infra-popliteal arteries is particularly challenging to treat due to the small caliber of the vessels and the heavy burden of atherosclerotic plaque. Percutaneous transluminal angioplasty is the conventional first-line approach and is advantageous due to its minimal invasiveness, repeatability, and cost-effectiveness but is limited by high rates of elastic recoil, dissection, and short- to mid-term re-stenosis. **Methods:** This review analyses the growing body of published and presented clinical data from multiple randomised controlled trials that have investigated the role of coronary drug-eluting stents in the treatment of infrapopliteal disease. **Results:** Coronary drug-eluting stents demonstrate superior primary patency compared with angioplasty and/or bare metal stenting alone but are limited to application in short-segment disease and have not been widely adopted due to the nature of the permanent implant. **Conclusions:** Newer devices like drug-eluting resorbable scaffolds are promising as they allow the restoration of vessel wall vasomotion without a residual foreign body and can be used to treat longer, complex lesions.

## 1. Introduction

Chronic limb-threatening ischaemia (CLTI) is the most severe manifestation of peripheral artery disease (PAD). It is characterised by tissue loss, gangrene, and/or chronic ulceration present for at least two weeks duration, and can result in limb amputation if left untreated. In 2015, PAD was estimated to affect more than 236 million people globally, with CLTI representing approximately 10% of those patients [1,2]. People suffering the disease are often afflicted by other disease states such as diabetes mellitus, heart, and renal failure. The overall prognosis is poor, with a 5-year mortality of 50–60% following an initial diagnosis of CLTI [3].

CLTI due to occlusive disease of the infrapopliteal arteries is challenging to treat due to the small caliber of vessels, long lesions, and the complexity of often heavily calcified plaque. An endovascular-first revascularisation strategy for infrapopliteal revascularisation is associated with superior amputation-free survival compared with bypass surgery and has become the first-line approach for most patterns of disease [4]. Historically, simple percutaneous transluminal angioplasty (PTA) has been used; however, it has been limited by high rates of restenosis which may require multiple reinterventions to maintain patency [5]. Following revascularisation, the clinicians’ objectives are to relieve pain, facilitate wound healing, and preserve a functional limb. Despite the high technical success rates of PTA, durability remains a limitation.

Multiple randomised controlled trials (RCTs) have demonstrated the efficacy of coronary drug-eluting stents (DESs) in improving primary patency, lowering immediate procedural residual stenosis and improving amputation rates compared with baremetal stents (BMSs) and/or PTA alone [5,6,7,8,9]. More recent devices, including drug-eluting resorbable scaffolds (DRSs), specialty, lithotripsy, and drug-coated balloons, as well as the Tack endovascular dissection repair system, designed specifically to treat post-PTA dissection, have sought to overcome the issue of residual foreign bodies that can add complexity to future endovascular procedures [10,11,12].

Guideline-directed medical therapy is recommended for all patients with CLTI. International guideline documents include the European Society of Cardiology and European Society for Vascular Surgery’s joint publication Guidelines on the Diagnosis and Treatment of Peripheral Artery Disease and the American Heart Association’s Guideline for the Diagnosis and Treatment of Peripheral Arterial Diseases [2,13,14]. This includes aggressive treatment with a long-term antiplatelet, low-dose direct oral anticoagulant, lipid-lowering therapy, antihypertensive, and strict glycaemic control. For patients with CLTI, the long-term use of a single antiplatelet agent is recommended to reduce the risk of major cardiovascular events [2]. Following endovascular lower limb revascularisation, patients who are not at high risk of bleeding are commonly treated with dual antiplatelet therapy for at least one month before continuing monotherapy long-term to reduce the risk of secondary cardiovascular and major adverse limb events. High-quality evidence on the optimum antithrombotic regimen post-lower limb revascularisation has yet to be published [2,15,16]. These recommendations should be offered in conjunction with counselling for lifestyle modifications including smoking cessation and preventative foot care, particularly in the context of diabetic neuropathy.

With regards to revascularisation strategy, the above guidelines do not make specific recommendations regarding an endovascular versus surgical approach for the treatment of infrapopliteal arterial disease but state that both options should be considered on an individual patient basis. Both above guidelines predate the recent BASIL-2 trial, which demonstrated the superiority of the best primary endovascular treatment over vein bypass in infrapopliteal disease [4]. In that study, 345 patients with previously untreated infrapopliteal CLTI were randomised to treatment with bypass or best endovascular treatment, which included PTA with or without drug-coated balloons, bare metal, or drug-eluting stents. The endovascular treatment group had lower rates of major amputation or death compared with the vein bypass group (63% vs. 53%, hazard ratio (HR) 1.35; *p* = 0.04), which was driven by fewer deaths in the best endovascular therapy group. Notably, they also had higher rates of reintervention compared with the bypass group (5% vs. 19%, risk ratio (RR) 0.27; 95% CI 0.13 to 0.55). However, given the minimally invasive and repeatable nature of PTA, this trial suggests that the majority of patients with arterial disease below the knee, with or without inflow disease, should be treated with endovascular therapy rather than bypass surgery first. The objective of this manuscript is to summarise the recent evidence, with a focus on new devices available for the treatment of below-knee atherosclerotic disease for patients with CLTI.

## 2. Treatment of Infrapopliteal Disease in CLTI

### 2.1. Balloon Angioplasty and Bare Metal Stents

PTA is the most common technique for below-the-knee endovascular revascularisation. Plaque disruption, medial and adventitial stretching, and localised dissection at the diseased segment permits restoration of luminal diameter and blood flow to the affected limb. PTA is minimally invasive, repeatable, and is an option for patients who are at high perioperative risk and unfit for general anesthesia. However, PTA is limited by high rates of elastic recoil, flow-limiting dissection, and restenosis [17]. In a meta-analysis, Romiti et al. found a 12-month primary patency rate of 58.1% after infrapopliteal PTA [18]. Giannopoulos et al. proposed an algorithm for infrapopliteal PTA that aimed to optimise each step of angioplasty and ultimately improve primary patency [17]. This included careful pre-procedure assessment of vessel calcification via intra- or extra-vascular ultrasound, use of a 1:1 or 1.1:1 balloon-to-artery diameter sizing, slow (1 atm every 5 to 10 s) and prolonged (at least 3 min) balloon inflations, and angiographic assessment of post-dilation dissection and recoil with multiple angles to avoid missing a significant problem.

Bare metal stenting has been used as a rescue method for these issues following angioplasty. A single randomised controlled study has compared outcomes between bare metal stenting and PTA for below-knee disease in CLTI [19]. This single-centre study randomised 38 limbs to treatment with angioplasty alone or bare metal stents (coronary balloon-expandable or self-expandible), finding no difference in overall survival (69.3% vs. 74.7%; *p* = 0.85), limb salvage (90% vs. 91.7%; *p* = 0.76), and primary (66% vs. 56%; *p* = 0.97) or secondary patency (79.5% vs. 64%; *p* = 0.81) at 12-month follow up. As such, BMSs are rarely used in infrapopliteal disease.

### 2.2. Drug-Eluting Stents

Coronary drug-eluting stents deliver targeted antiproliferative drug therapy directly to the blood vessel wall to reduce neointimal hyperplasia, negative remodelling, and instent re-stenosis. They have been used in the treatment of infrapopliteal artery disease for over a decade, with multiple randomised controlled trials demonstrating superior primary patency compared with PTA and/or BMSs [5,6,20]. Currently, they are often used as a bail-out strategy for post-angioplasty dissection, elastic recoil, or in the treatment of focal de novo disease. Sirolimus and its analogues inhibit the mechanistic target of rapamycin (mTOR) receptor that regulates smooth muscle proliferation. Inhibiting this pathway reduces neointimal hyperplasia and the risk of restenosis following vascular intervention. DESs coated in sirolimus analogues have exhibited superior primary patency over those coated with paclitaxel, which binds to and stabilises microtubules, inducing apoptosis and arresting mitosis [21,22,23,24].

The YUKON-BTK trial (NCT00664963) was the first published trial to compare DESs with BMSs. It randomised 161 patients with CLTI and intermittent claudication (IC) to endovascular treatment with polymer-free sirolimus-eluting stents or BMSs with a mean lesion length of 31 ± 9 mm [9]. The DES group had lower amputation rates of 2.6% vs. 12.2% (*p* = 0.03) and target vessel revascularisation rates of 9.2% vs. 20% (*p* = 0.06) compared with the BMS group. The DESTINY trial (NCT00510393) randomised 140 patients with Rutherford–Becker class 4 to 5 disease to treatment with a Xience V everolimus DES or BMS. This was associated with significantly improved 12-month primary patency for the DES group (85% vs. 54%; *p* < 0.01) but no difference in improvement in their Rutherford–Becker class (60% vs. 56%; *p* = 0.68) or 12-month mortality (82% vs. 84%; *p* = 0.96) [6]. In the ACHILLES trial (NCT00640770), 200 patients in Rutherford–Becker categories 3 to 5 were randomised to treatment with PTA or a sirolimus-coated DES. At 12 months, patients treated with DES had lower angiographic restenosis rates (22.4% vs. 41.9%; *p* = 0.02) and greater vessel primary patency (75% vs. 57.1%; *p* = 0.03) compared with those treated with PTA [5]. Lesions in the ACHILLES trial were more complex, as 81.3% of patients had chronic total occlusions and 15% of patients had heavily calcified lesions but the mean lesion length remained relatively short at 27 ± 21 mm. All three studies were performed on relatively short lesions with a notable absence of the long lesions that represent the majority of those seen in real world clinical practice, particularly in patients with diabetes and renal failure. This limitation has been consistent through most studies investigating DES.

The PADI trial (NCT00471289) compared a paclitaxel-coated DES with standard PTA with or without BMS in 74 limbs (73 patients with Rutherford–Becker class 4 to 6 disease) with infrapopliteal lesions up to 90 mm in length [8]. The 5-year amputation and event-free survival rate from the composite of major amputation or reintervention was higher in the DES arm (31.8 vs. 20.2%; *p* = 0.04 and 26.2 vs. 15.3%; *p* = 0.04) compared with PTA-BMS. Ten-year outcomes showed no significant difference between mortality in the paclitaxel DES group compared with the BMS group [20]. The IDEAS trial (NCT01517997) was a single-centre RCT that randomised 50 patients with Rutherford–Becker class 3 to 6 disease to treatment with a paclitaxel-coated balloon (PCB) angioplasty or a primary zotarolimus or sirolimus DES [7]. The mean lesion length was the longest in the trials, at 127 ± 46.5 mm. At 6-month follow-up, the DES group had lower immediate post-procedure stenosis (9.6% ± 2.2% vs. 24.8% ± 3.5% in the PCB arm; *p* < 0.0001), significantly lower binary restenosis rate (28% vs. 57.9%; *p* = 0.05), and no difference in target vessel revascularisation (7.7% vs. 13.6%; *p* = 0.65). Coronary DESs have been approved for use in infrapopliteal lesions in Europe and Australia but remain off-label in the United States.

The SAVAL trial (NCT03551496) was a prospective, multi-centre RCT that compared PTA with a paclitaxel-eluting nitinol stent designed specifically for the treatment of infrapopliteal CLTI. In the study, mean lesion lengths were 68.1 ± 35.2 mm in the DES group and 68.7 ± 49.2 mm in the PTA group [25]. However, at 12-month follow-up, the PTA treatment group had similar rates of primary patency compared with those treated with DES (76.0% vs. 68.0%; *p* = 0.86). The major adverse event-free rate was statistically indistinguishable between the two groups (95.3% vs. 91.6%; *p* = 0.04 for noninferiority) and, as such, neither the safety nor efficacy endpoints were met.

Multiple metanalyses have found DESs to be superior to conventional treatment with PTA and BMSs with respect to primary patency, freedom from target lesion revascularisation, major amputation, sustained Rutherford–Becker class improvement, and mortality in the infrapopliteal arteries (Table 1) [23,26,27,28,29]. However, DESs have seen limited adoption because of their pragmatic application to short lesions, concerns about burning bridges for future surgical intervention, and the nature of leaving behind a permanent metallic implant that may incur stent fracture, cause endothelial dysfunction and/or chronic inflammation that can lead to late restenosis and target lesion failure [30].

### 2.3. Drug-Coated Balloons

Drug coated balloons (DCBs) have also been applied in the treatment of infrapopliteal CLTI. A balloon coated in paclitaxel, an antiproliferative agent, is inflated to directly oppose against the vessel wall. This facilitates transient delivery of reservoirs of drug into the blood vessel wall with sustained effect [32]. Despite their theoretical advantages, DCBs have demonstrated mixed safety and effectiveness outcomes compared with PTA and DESs [7,32,33,34]. Single trials have shown disappointing results when DCBs have been compared with PTA; however, a 2022 meta-analysis (1479 patients in 10 studies), pooled results comparing treatments with DCB and PTA [35]. A total of 863 patients were treated with DCB and 616 with PTA. Patients treated with DCB had lower TLR (odds ratio (OR) 0.43; 95% CI 0.23 to 0.81; *p* < 0.01), decreased restenosis or occlusion (OR 0.42; 95% CI 0.19 to 0.93; *p* = 0.03), and late lumen loss (mean difference −0.52; 95% CI −0.84 to −0.20; *p* < 0.01) compared with PTA. There was no difference in all-cause mortality (OR 1.14; 95% CI 0.75 to 1.72; *p* = 0.54) or major amputation (OR 1.35; 95% CI 0.84 to 2.19; *p* = 0.22).

Two large randomised controlled trials have failed to demonstrate the superiority of DCB over PTA in the treatment of infrapopliteal artery disease. In the IN.PACT DEEP trial (NCT00941733), 357 patients were randomised in a 2:1 ratio to receive treatment with the paclitaxel-eluting Amphirion DCB (Medtronic, Minneapolis, MN, USA) or PTA [34]. At 6 months, DCBs were noninferior to PTA in the composite primary safety endpoint, which included all-cause death, major amputation, and CD-TLR (17.7% vs. 15.8%; *p* = 0.02 for noninferiority), but were associated with a trend towards major amputation at 1 year (8.8% vs. 3.6%; *p* = 0.08). At 12 months, there was no difference in CD-TLR (9.2% vs. 13.1%; *p* = 0.29) or late lumen loss (0.61 ± 0.78 mm vs. 0.62 ± 0.78 mm; *p* = 0.95); thus, the primary efficacy endpoints were not met. In the LUTONIX BTK trial (NCT01870401), 442 patients with Rutherford–Becker category 3 to 5 disease were randomised in a 2:1 ratio to treatment with Lutonix paclitaxel-eluting DCBs (Becton Dickinson, Franklin Lakes, NJ, USA) or PTA [36]. The 12-month data indicated no significant difference in freedom from above-ankle amputation, target lesion occlusion, or CD-TLR between DCB and PTA treatment groups (60.3% vs. 60.9%; 95% CI 0.108–0.101; *p* = 0.54). Neither trial showed an advantage of DCB use over PTA.

The IN.PACT BTK trial (NCT02963649) was a prospective, multicentered randomised pilot study that aimed to evaluate the safety and effectiveness of the IN.PACT 0.14 paclitaxel DCB (Medtronic, Minneapolis, MN, USA) in treating infrapopliteal CLTI [32]. In that study, 50 patients with Rutherford–Becker 4 and 5 disease were randomised to treatment with DCBs (*n* = 23) or PTA (*n* = 27). Patients had a mean lesion length of 215.4 ± 83.8 mm. This study was small and not statistically powered for reintervention, and it did not meet its effectiveness endpoint of late lumen loss at 9 months on conventional assessment (DCB 0.89 ± 0.77 mm vs. PTA 1.31 ± 0.72 mm; *p* = 0.07), although it did on subsegmental analysis (DCB 0.592 ± 0.944 mm vs. PTA 1.260 ± 0.810 mm; *p* = 0.02). Inconsistent and variable results that have failed to translate to large-scale RCTs have limited the widespread application of these devices in the primary treatment of infrapopliteal disease.

Two sirolimus-coated balloons, the Selution SLR DEB (MedAlliance SA, Nyon, Switzerland) and the MagicTouch PTA DEB (Concept Medical, Tampa, FL, USA), have been approved for investigational use by the US Food and Drug Administration. Sirolimus has lower lipophilicity when compared to paclitaxel and both devices aim to rectify this issue by applying phospholipid technology to improve drug bioavailability [37]. The Selution SLR DCB (MedAlliance SA, Nyon, Switzerland) uses polylactic co-glycolic acid micro-reservoirs and a cell-adherent technology phospholipid layer to prevent wash-off and improve absorption of the reservoirs containing sirolimus into the vessel wall. The MagicTouch DCB (Concept Medical, Tampa, FL, USA) uses sub-micron-sized sirolimus particles coated in a phospholipid carrier to minimise drug loss during transit and improve diffusion and retention into the wall [38]. Tang et al. were among the first to assess the use of the Selution SLR drug-coated balloon in patients with infrapopliteal CLTI [39]. In a single-armed, non-randomised pilot study, 25 patients (33 lesions, mean lesion length 19.1 ± 11.1 mm) with Rutherford–Becker category 5 disease were treated with the Selution SLR DCB, with sirolimus administered at a dose of 1 µg/mm^2^ to obtain a sustained effect for up to 90 days. At 6-month follow-up, primary tibial patency was 81.5% and freedom from CD-TLR was 83.3%. These outcomes were sustained at 12 months, which is a promising result. Larger randomised trials including SELUTION4BTK (NCT05055297), LIMES (NCT0477230), and FUTURE-BTK (NCT04511247) the latter two of which are to investigate the MagicTouch PTA Sirolimus DCB, are currently in the recruitment phase [40,41,42]. Hopefully, results from current single-centre studies can be replicated on a larger scale.

### 2.4. Tack Endovascular System

The true prevalence of dissection following infrapopliteal PTA is difficult to determine. However, it has been conservatively estimated to occur in 15 to 30% of cases and is associated with early occlusion and late restenosis, which may require repeat endovascular intervention [10,43].

The Tack endovascular system (Intact Vascular, Wayne, PA, USA) comprises a single device containing four self-expanding nitinol implants and was specifically designed to treat dissection following balloon angioplasty. The device was created with open geometry to exert a low outward radial force with minimal metal contact with the vessel wall and can self-size to vessel diameters of 1.5–4.5 mm [10]. The TOBA II BTK trial (NCT02942966) was a prospective, multi-centre, single-arm study that analysed 248 infrapopliteal lesions in 233 patients [11]. In total, 341 dissections were identified following angioplasty. Primary endpoints were major adverse limb events (MALEs) and all-cause perioperative death (POD). The mean lesion length was 80 ± 49 mm and patients received at least one Tack implant (up to 16). Freedom from MALEs at 24 months and POD at 30 days was 92.2% and 24-month freedom from CD-TLR was 73.6%. Recently published 36-month outcomes showed a 93.9% target limb salvage, 69.6% freedom from CD-TLR, and improvements in patient-reported quality of life and mobility [44]. The study demonstrates the sustained efficacy and safety of the device in a group of patients known to have poor outcomes but who are rarely studied, suggesting that the device may have an important role in focal dissection repair following PTA.

### 2.5. Drug-Eluting Bioresorbable Scaffolds

Drug-eluting bioresorbable scaffolds (DRSs) were designed to overcome the issues associated with BMSs and DESs related to permanent metallic foreign bodies. DRSs provide the benefits of an antiproliferative drug coating to minimise neointimal hyperplasia with structural support to overcome elastic recoil, without the permanency of a stent. The first generation of bioresorbable scaffolds (Absorb, Abbot Vascular, Santa Clara, CA, USA) showed promising results in reducing restenosis, rates of clinically driven target lesion revascularisation, and improving primary patency in infrapopliteal arteries [30,45].

In the first study to evaluate the long-term outcomes of the Absorb DRS in infrapopliteal arteries, 61 lesions (mean length 20.1 ± 10.8 mm) in 48 patients with Rutherford–Becker 3 to 5 infrapopliteal CLTI were treated [45]. Mean follow-up occurred at 35.2 ± 20.4 months, following which 22 patients (45.8%) had died, consistent with the natural history of CLTI. For those who reached follow-up, the limb salvage rate was 100%. Complete wound healing occurred in 87.2% of patients treated for tissue loss. Binary restenosis was detected in only 11 patients (15.5%) with scaffolds over the follow-up period, with all but one case being in the moderate range between 50–75% restenosis.

Ipema et al. conducted the first metanalysis assessing the use of coronary DRSs in infrapopliteal arterial disease, examining five studies that used three first-generation DRSs—the Absorb BVS (Abbott Vascular, Santa Clara, CA, USA), the Absorbable Metal Stent (Magic, Biotronik, Berlin, Germany), and the Bioimus A9-eluting stent (BES, BioMatrix Flex, Biosensor International, Newport Beach, CA, USA) [30]. They found a 12-month primary patency of 90% (95% CI 0.84 to 0.95), a 12-month freedom from clinically driven target lesion revascularisation rate of 96% (95% CI 0.91 to 0.99), and 12-month limb salvage rate of 97% (95% CI 0.91 to 0.99). These scaffolds were ultimately withdrawn from the market due to association with increased target-vessel myocardial infarction following treatment of coronary artery stenosis [46]. Despite this, DRSs have promising applications in the treatment of infrapopliteal disease as full degradation through bioresorption allows the potential return of vasomotion, vessel wall remodeling, and absence of a stent that may impede future intervention.

The LIFE-BTK trial (NCT04227899) was a multicentre, single-blind, randomised controlled trial designed to assess the safety and efficacy of the novel Esprit BTK scaffold (Abbot Vascular, Santa Clara, CA, USA) in treating infrapopliteal lesions in patients with CLTI. The Espirit BTK scaffold is made of poly-L-lactic acid (PLLA) coated with poly D,L-lactide (PDLLA) surface polymer that provides a controlled release of everolimus at a concentration of 100 µg/mm^2^, similar to that of a Xience DES. The scaffold is biodegradable through hydrolysis and is completely resorbed by 36 months [47]. The device has thinner struts (99 µm–120 µm depending on scaffold diameter) and is produced in longer lengths than the coronary model.

In the study, 261 patients with infrapopliteal CLTI (Rutherford–Becker 4 and 5) were randomised in a 2:1 ratio to treatment with an everolimus-eluting DRS or angioplasty. The primary efficacy endpoint was a composite consisting of freedom from above-ankle amputation of the target limb, total occlusion of the target vessel, clinically driven revascularisation, and binary restenosis at 12 months [12]. The mean lesion length was 43.8 ± 31.8 mm in the DRS group and 44.8 ± 29.1 mm in the angioplasty group. Immediate technical success occurred in 91% of patients in the DRS group and 70% of the angioplasty group, with 5 patients requiring bailout stenting.

Everolimus-eluting DRSs were superior to angioplasty in the composite primary efficacy endpoint (74% vs. 44%; *p* < 0.01 for superiority) [31]. Further, treatment with the DRSs was non-inferior to angioplasty with respect to the primary safety endpoint of freedom from MALEs at 6 months and POD (*p* < 0.0001 for noninferiority).

LIFE-BTK was the first RCT to assess the safety and efficacy of DRSs in patients with infrapopliteal CLTI. These results demonstrate the superiority of DRSs over angioplasty in several important domains (Table 2). Though the Esprit BTK scaffold is currently investigational only, approval by international regulatory bodies could signal a shift towards a new standard of care.

Other DRSs are currently being investigated for safety and efficacy, including the sirolimus-eluting MOTIV bioresorbable scaffold (REVA Medical, San Diego, CA, USA) and the MAGNITUDE bioresorbable DRSs (R3 Vascular, Santa Clara, CA, USA). The MOTIV DRS uses a Tyrocore scaffold, a novel polymer derived from tyrosine, which is visible on fluoroscopy. Strut thickness ranges from 95 to 115 µm. The MOTIV BVS BTK pilot study (NCT03987061) was a prospective, single-arm, multicentre study that investigated 76 MOTIV scaffolds in 60 limbs over 36 months. The mean lesion length was 29.46 mm and the study included treatment of both primary de novo lesions and restenotic lesions (*n* = 37) as well as areas of flow-limiting dissection post-PTA or restenosis after PTA of a longer lesion (*n* = 23). The preliminary 6-month results showed 99% technical success, 90% primary patency, and a CD-TLR rate of 3% [48]. A global MOTIV BTK RCT is currently underway with primary efficacy endpoints being freedom from above-ankle amputation, CD-TLR, and target lesion occlusion at 6 months, and the primary safety endpoint is freedom from all-cause POD and MALEs of the index limb involving the infrapopliteal arteries at 30 days [49].

The MAGNITUDE DRS (R3 Vascular, Santa Clara, CA, USA) is a sirolimus-eluting PLLA polymer scaffold that is coated in PDLLA [50]. It has a strut thickness of 98 µm and high radial strength and is available in lengths up to 58 mm. Molecular weight is reduced by 95% at 18 months. The RESOLV I trial (NCT04912323) was a prospective, single-arm, multi-centre, first-in-humans study to assess the use of the Magnitude DRS in up to 50 patients with Rutherford–Becker category 3 to 5 infrapopliteal disease. The mean lesion length was 34.6 ± 15.4 mm. Preliminary 6-month data were presented demonstrating that all patients (*n* = 28/28) met the primary safety endpoints of freedom from MALEs, defined as above-ankle amputation in the index limb or major re-intervention within 180 days, and POD within 30 days. The primary efficacy endpoints of angiographic primary patency and freedom from TLR at 6 months were met in 93% of lesions (*n* = 27/29). A large, global RCT to assess the use of MAGNITUDE DRS in infrapopliteal lesions is planned. The results of both the MAGNITUDE and MOTIV studies are highly anticipated.

## 3. Future Directions

The outcomes from the LIFE-BTK trial may represent a step towards developing improved endovascular treatment options for patients with CLTI in this complex anatomical region. One criticism of this study was that participants had shorter lesions than would usually be encountered in clinical practice; however, subgroup analysis found that patients with the longest tercile lesions benefited the most from scaffold over PTA. Now that the safety and efficacy of the Esprit DRS have been demonstrated, replication in a larger clinical population registry with longer infrapopliteal lesions may provide insights.

Furthermore, a cost and quality-of-life analysis of DRSs compared with PTA and DESs would be a valuable addition to the space, as healthcare payers struggle with the increasing costs of treating this burgeoning patient population. PTA has been established as a cost-effective strategy for the treatment of infrapopliteal CLTI compared with stenting, though it is possible that with new DRSs, avoiding re-hospitalisation for repeat endovascular interventions to treat restenosis associated with PTA may provide a net benefit [51]. A prospective cost analysis is planned to be completed based on data from LIFE-BTK.

There has been no randomised controlled trial to directly compare DRSs with DESs in the treatment of infrapopliteal CLTI. As previously described, the theoretical benefits include the absence of a permanent metallic implant that drives chronic inflammation and neointimal hyperplasia. In the coronary circulation, treatment with a DES currently remains the gold standard due to very low rates of target lesion failure [52]. New third-generation coronary DRSs are currently being evaluated for safety and efficacy. These have been designed with thinner struts, like the DREAMS 3G (BIOTRONIK, Berlin, Germany), a sirolimus-eluting magnesium scaffold that is being investigated in the first-in-humans BIOMAG-I trial (NCT04157153) [53,54]. The 12-month outcomes have recently been published, with in-scaffold late lumen loss at 6 and 12 months of 0.21 mm (SD 0.31) and 0.24 ± 0.36 mm, respectively [54].

Beyond DRSs, other novel technologies for infrapopliteal CLTI treatment include intravascular lithotripsy (IVL), which uses emitters mounted on a traditional angioplasty balloon catheter that create microfractures within superficial and deep calcium without liberating emboli [55]. The DISRUPT BTK study (NCT02911623) was a single-arm feasibility study that evaluated the use of the Shockwave Peripheral lVL system (Shockwave Medical, Santa Clara, CA, USA) in 20 patients with moderate to severe infrapopliteal artery calcification, 16 of whom had CLTI [56]. At 30-day follow-up, all 19 patients who were treated with IVL had ≤50% residual stenosis, with a mean diameter stenosis of 26.2%. There were no major adverse events, defined as death, myocardial infarction, revascularisation, or major amputation, and no episodes of distal embolisation, perforation, or thrombus formation. The ongoing DISRUPT PAD BTK II (NCT05007925) is a large global RCT aiming to evaluate IVL in 250 patients with infrapopliteal CLTI [52]. This trial is still in its recruitment phase; however, its success would offer a possible solution for severe medial calcification, which is associated with an increased risk of amputation and mortality and remains an area of unmet need [57].

## 4. Conclusions

There have been significant advancements in the approach to treating infrapopliteal CLTI over the past decade, with a first-line endovascular approach being preferred.

Although simple balloon angioplasty remains the standard due to its safety, cost-effectiveness, and repeatability, it is limited by high rates of restenosis and flow-limiting dissection. The Tack endovascular system is a valuable rescue device post-PTA dissection but still results in a permanent metallic implant in small-calibre arteries. The use of coronary sirolimus-eluting DESs has shown encouraging results, with excellent primary patency compared with PTA and BMSs, but they are limited by their short length, which complicates applicability to long disease segments that represent the majority of infrapopliteal CLTI, as well as affecting cost. Novel DRSs pose a solution to some of these issues by providing an antiproliferative agent before completely resorbing, eliminating the risk of chronic inflammation and late failure associated with permanent implants.

Devices like the Esprit BTK bioresorbable scaffold and similar next-generation devices may herald a new generation of treatment options to overcome the historical challenges of treating long occlusive segment disease.

## Figures and Tables

**Table 1:** Summary of randomised controlled trials and their primary endpoint(s).

Trial	Drug-Eluting Stent or Scaffold	Control	Participants in Drug-Eluting Device Group *n* (%)	Mean Lesion Length (mm)	Follow-Up Interval	Primary Endpoint(s)
YUKON BTK [9]	Sirolimus DES	PTA + BMS	82/161 (51)	31 ± 9	DES: 1005 ± 139 days BMS: 1027 ± 123 days	Event-free survival * DES: 65.8% vs. PTA + BMS: 44.6%; *p* = 0.02
DESTINY [6]	Everolimus DES	PTA + BMS	74/140 (53)	DES: 15.9 ± 10.2 PTA + BMS: 18.9 ± 10.0	12 months	12-month primary patency (absence of ≥50% restenosis) DES: 85% vs. PTA + BMS 54%; *p* = 0.0001
ACHILLES [5]	Sirolimus DES	PTA	99/200 (50)	26.9	12 months	12-month in-segment binary restenosis by quantitative angiography DES: 22.4 vs. PTA: 41.9%; *p* = 0.02
PADI [8]	Paclitaxel DES	PTA ± BMS	DES: 74/140 limbs in 137 patients	DES: 21.1 ± 19.3 PTA ± BMS: 23.1 ± 21.8	6 months, 12 months, and 24 months	6-month primary patency (≤50% stenosis on CT angiography) DES: 48.0 vs. PTA ± BMS: 31.5%; *p* = 0.10
IDEAS [7]	Paclitaxel DES	Paclitaxel DCB	DES: 30 arteries in 27 limbs PCB: 25 arteries in 25 limbs	DES: 127 ± 46.5 PCB: 148 ± 56	6 months	6-month binary restenosis (>50%) DES: 28% vs. PCB: 57.9% *p* = 0.05
SAVAL [25]	Nitinol (self-expanding) paclitaxel DES	PTA	130/201 (65)	DES: 68.1 ± 35.2 PTA: 68.7 ± 49.2	1, 3, 6, and 12 months	Efficacy: 12-month primary vessel patency DES: 68.0% vs. PTA: 76.0; *p* = 0.86 Safety: 12-month MAE-free rate DES: 91.6% vs. PTA: 95.3%; *p* = 0.43
LIFE-BTK [31]	Everolimus-eluting DRS	PTA	173/261 (66)	DRS: 43 ± 31.8 PTA: 44.8 ± 29.1	12 months	Efficacy: 12-month freedom from: above-ankle amputation of target limb, CD-TLR, and binary restenosis BRS: 74% vs. PTA: 44%; *p* < 0.001 Safety: freedom from MALEs 6 months and POD BRS: 165/170 and PTA: 90/90; *p* < 0.001 noninferiority

Abbreviations: DES: drug-eluting stent; BMS: bare metal stent; PTA: percutaneous transluminal angioplasty; CD-TLR: clinically driven target lesion revascularisation; DRS: drug-eluting bioresorbable scaffold; DCB: drug-coated balloon; MAE: major adverse event; MALEs: major adverse limb events; POD: perioperative death. * Defined as freedom from target limb amputation, target vessel revascularization, myocardial infarction, and death.

**Table 2:** Benefits and limitations of devices used to treat infrapopliteal CLTI.

Device	Benefits	Limitations
Percutaneous angioplasty	Cost-effective Repeatable No permanent implant	High rates of elastic recoil and dissection High rates of restenosis
Bare metal stent	Rescue following failed angioplasty	Permanent metallic implant Risk of stent fracture
Drug-coated balloon	No permanent implant	Mixed evidence for efficacy
Drug-eluting stent	Superior primary patency compared with angioplasty and bare metal stenting	Permanent metallic implant Risk of stent fracture Limited to application in short lesions
Tack endovascular system	Only device available for use as rescue post-dissection	Permanent metallic implant
Drug-eluting bioresorbable scaffold	No permanent implant Superior primary patency compared with angioplasty Restoration of wall vasomotion	Investigational device only Cost analysis pending

## Data Availability

Data sharing not applicable. No new data were created or analyzed in this study. Data sharing does not apply to this article.
